# Clinical features of pneumatosis intestinalis induced by alpha- glucosidase inhibitor in patients with type 2 diabetes mellitus: a single center retrospective study

**DOI:** 10.3389/fendo.2025.1470523

**Published:** 2025-02-07

**Authors:** Guanlin Wu, Weiheng He, Huimin Rao, Lin Lu, Xinran He, Xuewen Hou

**Affiliations:** ^1^ School of Clinical Medicine, Shanghai University of Medicine & Health Sciences, Shanghai, China; ^2^ Department of Radiology, People’s Hospital of Ningxia Hui Autonomous Region, Yinchuan, Ningxia, China; ^3^ Department of Gastrointestinal Surgery, People’s Hospital of Ningxia Hui Autonomous Region, Yinchuan, Ningxia, China; ^4^ Department of Radiology, The First Dongguan Affiliated Hospital, Guangdong Medical University, Dongguan, Guangdong, China

**Keywords:** type 2 diabetes, acarbose, side effects, pneumatosis intestinalis, clinical features

## Abstract

**Purpose:**

Pneumatosis intestinalis (PI) is a rare but significant side effect associated with the use of alpha-glucosidase inhibitor (αGI) in the treatment of diabetes. This study aims to analyze the clinical features of PI induced by αGIs in patients with type 2 diabetes mellitus.

**Methods:**

We conducted a retrospective analysis of patients diagnosed with PI between January 2018 and December 2023. Data collected included demographic characteristics, clinical symptoms and signs, laboratory findings, imaging results, endoscopic manifestations, treatments, and outcomes. Clinical characteristics were compared between patients who used acarbose and those who did not.

**Results:**

A total of 48 patients with PI were included in the study, of whom 22 had used acarbose and 26 had not. The acarbose taken group was significantly older than the acarbose untaken group. Additionally, the prevalence of coronary heart disease and hypertension was markedly higher in patients taking acarbose. Importantly, total bilirubin levels were lower in those with PI who were on acarbose therapy.

**Conclusion:**

Our findings highlight the need for increased vigilance regarding the potential development of PI in older diabetic patients with cardiovascular conditions following αGI administration. Timely intervention is crucial to prevent adverse outcomes. This study offers valuable insights for the future management of αGI in diabetes treatment.

## Introduction

Pneumatosis intestinalis (PI), also referred to as pneumatosis cystoides intestinalis, is classified as a gastrointestinal disorder that describes the accumulation of intramural gas in the intestines, which was first documented by Du Vernoi in 1783, marking a significant milestone in the understanding of gastrointestinal pathology (1). PI is considered to be a rare disease, with reported incidence rates of approximately 0.03% in the general population (2). The classification of PI can be divided into two main types: idiopathic type, which accounts for about 15% of cases and is characterized by the presence of cystic air pockets indicative of a chronic, benign idiopathic etiology; and secondary type, which constitutes approximately 85% of cases and is defined by specific radiological findings of linear, microvesicular, or circumferential intramural gas resulting from a variety of predisposing factors (3, 4).

Working as a class of hypoglycemic agent, alpha glucosidase inhibitor (αGI) is the common prescription drug for treatment of type 2 diabetes mellitus, which non systematically slows down carbohydrate digestion and reduces postprandial hyperglycemia by delaying carbohydrate absorption in the small intestine through antagonism, or by antagonizing the dose-dependent inhibitory effect of alpha-glucosidase to delay the absorption of hydrates in the small intestine (5). However, the use of αGI is often associated with gastrointestinal side effects, which are the most frequently reported adverse reactions and include symptoms such as abdominal pain, bloating, and diarrhea (6, 7). These gastrointestinal side effects are a primary reason for some patients with type 2 diabetes discontinue their αGI treatment (8).

Interestingly, PI has been noted as a rare side effect resulting from the use of αGI in the treatment of diabetes, a recent study utilized data from the US Food and Drug Administration Adverse Event Reporting System to identify safety signals indicating a significant association between αGI and PI, revealing a markedly higher reporting odds ratio for PI among αGI, particularly for voglibose and miglitol, while no safety signals were detected for other anti-hyperglycemic drug classes, thereby highlighting the potential life-threatening risk associated with αGI use in patients with carbohydrate-rich diets (9). There are also several case reports emerging over the past decade documenting this association. For example, S. Tanabe et al. successfully treated a patient who developed pneumoperitoneum attributed to αGI use, highlighting the potential complications of this medication (10). Similarly, A. Rottenstreich et al. reported a rare case of benign PI accompanied by portal venous gas and pneumoperitoneum induced specifically by the drug acarbose (11). Furthermore, A. Police et al. published a case report detailing PI induced by αGI in the setting of sigmoid volvulus in a diabetic patient (12). Notably, S. Otsuka et al. described a 59-year-old lung transplant recipient who developed asymptomatic PI after four years of treatment with alpha-glucosidase inhibitors for diabetes, highlighting the need for physicians to recognize this rare adverse drug reaction and the importance of immediate discontinuation of αGI therapy with subsequent conservative management in such patients (13). These cases underscore the necessity for clinicians to exercise increased vigilance regarding αGI-induced PI in their practice. However, despite these individual reports, there remains a notable absence of systematic studies investigating the clinical characteristics of PI induced by αGI in patients with diabetes.

In addition to focusing on diabetes itself, we also need to pay attention to the impact of other accompanying diseases on the progress of diabetes like cardiovascular diseases. Type 2 diabetes is closely linked to an increased risk of cardiovascular disease (14). Hyperglycemia, insulin resistance, and associated metabolic disturbances characteristic of type 2 diabetes contribute to the development of atherosclerosis and endothelial dysfunction, leading to cardiovascular complications (15). Patients with type 2 diabetes often exhibit comorbid conditions such as hypertension and dyslipidemia, which further exacerbate the risk of cardiovascular disease (16). Moreover, the inflammatory state observed in diabetes may promote vascular damage and plaque formation in arteries (17). Consequently, individuals with type 2 diabetes are significantly more prone to events such as heart attacks and strokes, making it essential for healthcare providers to emphasize cardiovascular risk management in diabetic patients. Therefore, we need to pay close attention to the potential impact of other conditions on the occurrence of PI in patients.

This study aims to fill this gap in the literature by exploring the clinical features of PI induced by αGI specifically in patients with type 2 diabetes mellitus. The findings of this research will provide valuable guidance for the management of this rare but concerning side effect, ultimately enhancing patient care and treatment outcomes in this population.

## Materials and methods

### Study design and patients

This retrospective study was performed at People’s Hospital of Ningxia Hui Autonomous Region (a tertiary comprehensive hospital), China. The study conforms to Declaration of Helsinki. Our institutional review board approved this study. The requirement for informed consent was waived because of the retrospective nature of the study.

We searched electronic medical database of our hospital from January 2018 to December 2023. Diabetes, acarbose, and PI were input as keywords for the study. PI was defined as the presence of gas within the wall of the gastrointestinal tract. The inclusion criteria for patients in this study comprised a diagnosis of PI confirmed through abdominal computed tomography (CT) and/or endoscopic examination, alongside the availability of complete clinical data for all participants. Furthermore, a comprehensive review of the patients’ medical histories was conducted to categorize those with a history of long-term acarbose use into the acarbose taken group, while other patients with PI were assigned to the acarbose untaken group. Patients in the acarbose taken group who have long-term use of antibiotics, immunosuppressants, analgesics, and other medications that may cause PI should be excluded.

Demographic characteristics (gender, age, height, weight, BMI, temperature, heart rate, respiratory rate, systolic blood pressure, diastolic blood pressure, history of coronary heart disease, history of hypertension, history of chronic obstructive pulmonary disease, and history of abdominal surgery), clinical symptoms and signs (celialgia, ventosity, emesis, fever, hematochezia, and peritoneal irritation syndrome), laboratory findings (white blood cell count, neutrophil count, red blood cell count, platelet count, lymphocyte count, eosinophil count, basophil count, C-reactive protein, lactate dehydrogenase, alanine aminotransferase, alkaline phosphatase, total bilirubin, albumin, globulin, serum creatinine, and blood urea nitrogen), abdominal CT findings (Segment of gastrointestinal tract involved, intestinal obstruction, intestinal dilatation, intestinal wall thickening, intestinal wall edema, mesenteric edema, pneumoperitoneum, peritonitis, ascites, hepatic portal and mesenteric venous gas, superior and inferior mesenteric artery diameters, superior mesenteric arteriosclerosis, inferior mesenteric arteriosclerosis, and mesenteric venous thrombosis), endoscopic manifestations, treatments, and outcomes of patients were recorded and analyzed.

### Statistical analysis

Data for categorical variables were expressed as frequency rates and percentages and the differences between the two groups were compared using Chi-square test. Continuous variables are described in mean ± standard deviation or median (interquartile range) and the differences between the two groups were compared using two independent samples t-test or Wilcoxon rank sum test. There is a statistically significant difference when the *P*-value is less than 0.05. All data were analyzed by using GraphPad Prism 9.0 (San Diego, USA) and SPSS 26.0 (Chicago, USA).

## Results

### Baseline characteristics of patients

As shown in **Table 1**. A total of 48 PI patients were enrolled in this study. Of these patients, 22 PI patients had acarbose usage (200mg with meals for more than 10 years) and 26 patients had not. The age and respiratory rate of patients in the acarbose taking group were higher than those in the non-taking group (P < 0.05). Coronary heart disease and hypertension were much more frequent in the acarbose taking group (P < 0.05). With regard to clinical symptoms, emesis was more frequent in patients taking acarbose (40.9% versus 11.5%, P = 0.042). There were no significant differences in other variables between the two groups (P > 0.05).

**Table 1:** Clinical characteristics of patients with pneumatosis intestinalis induced by acarbose.

Parameters	Acarbose taken (n=22)	Acarbose untaken (n=26)	P value
**Age** (years)	74.7 ± 10.0	63.5 ± 15.1	**0.006**
**Sex**			0.148
**Female**	13 (59.1%)	9 (34.6%)	
**Male**	9 (40.9%)	17 (65.4%)	
**Height** (cm)	164.6 ± 7.5	167.7 ± 7.7	0.172
**Weight** (Kg)	63.6 ± 7.7	66.1 ± 11.7	0.232
**BMI** (kg/m²)	23.6 ± 3.2	23.4 ± 3.3	0.908
**Temperature** (°C)	36.8 ± 0.6	36.5 ± 0.5	0.141
**Heart rate** (Beats/minute)	84.5 ± 19.8	84.3 ± 13.2	0.849
**Respiratory rate** (Times/minute)	21.8 ± 5.3	18.8 ± 4.1	**0.042**
**Systolic blood pressure** (mmHg)	129.9 ± 18.5	123.3 ± 13.7	0.080
**Diastolic blood pressure** (mmHg)	76.0 ± 9.9	77.2 ± 11.0	0.896
**Comorbidities**			
**Coronary heart disease**	5 (22.7%)	0 (0%)	**0.015**
**Hypertension**	13 (63.6%)	4 (15.4%)	**0.001**
**COPD**	2 (9.1%)	2 (7.7%)	0.862
**Clinical symptoms and signs**			
**Celialgia**	15 (68.2%)	16 (61.5%)	0.765
**Ventosity**	9 (40.9%)	7 (26.9%)	0.366
**Emesis**	9 (40.9%)	3 (11.5%)	**0.042**
**Fever**	1 (4.5%)	0 (0%)	0.458
**Hematochezia**	0 (0%)	1 (3.8%)	0.542
**Peritoneal irritation sign**	6 (27.3%)	4 (15.4%)	0.478
**History of abdominal operation**	8 (36.4%)	8 (30.8%)	0.764

BMI, body mass index; COPD, chronic obstructive pulmonary disease. The bold values indicating P value < 0.05.

### Baseline laboratory findings

Our study found that total bilirubin level was lower in PI patients taking acarbose, and there were no significant statistical differences in other laboratory parameters between the two groups (**Table 2**).

**Table 2:** Comparison of laboratory test results of patients with pneumatosis intestinalis between the acarbose taken and untaken group.

Parameters	Acarbose taken (n=22)	Acarbose untaken (n=26)	P value
**White blood cell count** (×10^9^ /L)	8.4 ± 5.2	8.2 ± 4.9	0.817
**Neutrophil count** (×10^9^ /L)	6.4 ± 5.4	6.3 ± 4.8	0.914
**Red blood cell count** (×10^12^/L)	4.5 ± 0.6	4.6 ± 0.6	0.118
**Platelet count** (×10^9^ /L)	200.4 ± 68.5	195.6 ± 32.3	0.626
**Lymphocyte count** (×10^9^ /L)	1.1 ± 0.7	1.4 ± 0.9	0.181
**Eosinophil count** (×10^9^ /L)	0.09 ± 0.08	0.09 ± 0.07	0.575
**Basophil count** (×10^9^ /L)	0.011 ± 0.009	0.013 ± 0.011	0.569
**C-reactive protein** (mg/L)	33.3 ± 29.2	37.4 ± 30.4	0.798
**Lactate dehydrogenase** (U/L)	198.5 ± 58.0	208.8 ± 116.0	0.357
**Alanine aminotransferase** (U/L)	18.8 ± 7.4	29.4 ± 11.8	0.255
**Alkaline phosphatase** (U/L)	62.6 ± 21.3	68.1 ± 27.0	0.541
**Total bilirubin** (μmol/L)	12.5 ± 3.3	17.7 ± 8.1	**0.005**
**Albumin** (g/L)	36.9 ± 5.7	38.7 ± 7.9	0.534
**Globulin** (g/L)	23.8 ± 4.7	23.1 ± 4.8	0.618
**Serum creatinine** (μmol/L)	70.7 ± 31.0	86.6 ± 44.4	0.096
**Blood urea nitrogen** (mmol/L)	6.8 ± 3.5	7.1 ± 3.9	0.735

The bold values indicating P value < 0.05.

### Abdominal CT findings

**Figure 1:**
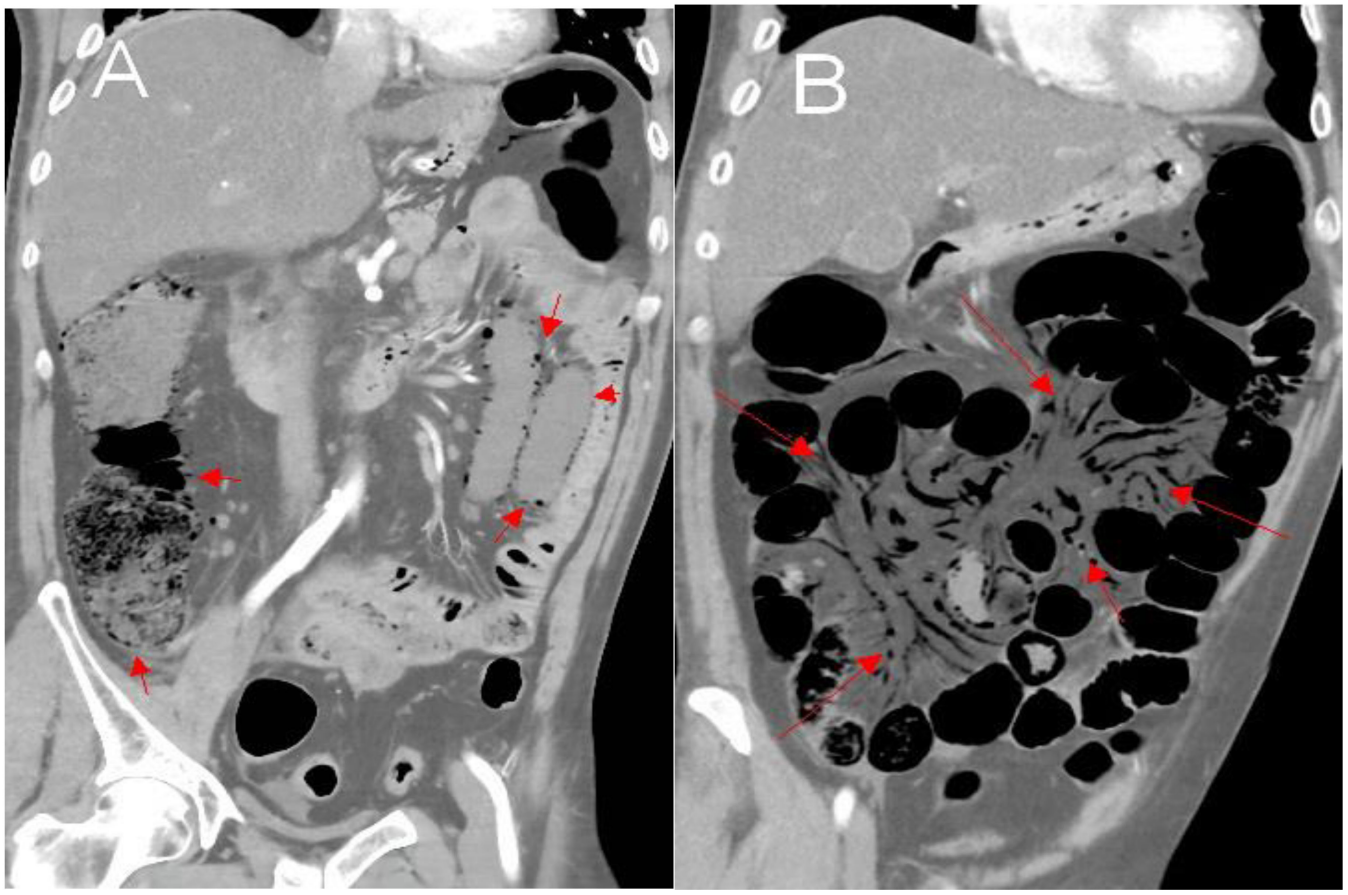
Abdominal CT revealed air in multiple small cysts within the wall of the large and small bowel and numerous mesenteric venous gas, with free air scattered in peritoneal cavity **(A, B)**.

As shown in **Figure 1**. Numerous submucosal air-filled cysts were observed in the wall of the large and small bowel, with numerous mesenteric venous gas and free air scattered in peritoneal cavity (**Figures 1A, B**). PI patients who took acarbose had higher incidence of inferior mesenteric arteriosclerosis than PI patients who did not take acarbose (*P* = 0.044), and the other abdominal CT features were not statistically significant between the two groups (**Table 3**).

**Table 3:** Comparison of abdominal CT findings of patients with pneumatosis intestinalis between the acarbose taken and untaken group.

Parameters	Acarbose taken (n=22)	Acarbose untaken (n=26)	P value
**Segment of gastrointestinal tract involved**			
Stomach	0 (0%)	1 (3.8%)	0.542
Small bowel	8 (36.3%)	8 (30.7%)	0.458
Colon	18 (81.8%)	20 (76.9%)	0.479
**Intestinal obstruction**	7 (31.8%)	7 (26.9%)	0.477
**Intestinal dilatation**	5 (22.7%)	6 (23.1%)	0.625
**Intestinal wall thickening**	22 (100%)	25 (96.2%)	0.542
**Intestinal wall edema**	7 (31.8%)	5 (19.2%)	0.341
**Mesenteric edema**	5 (22.7%)	6 (23.1%)	0.977
**Pneumoperitoneum**	12 (54.5%)	10 (38.5%)	0.384
**Peritonitis**	5 (22.7%)	6 (23.1%)	0.977
**Ascites**	7 (31.8%)	9 (34.6%)	0.542
**Hepatic portal and mesenteric venous gas**	6 (27.3%)	2 (7.7%)	0.077
**Superior mesenteric artery diameter** (mm)	7.7 ± 1.0	7.5 ± 1.3	0.582
**Inferior mesenteric artery diameter** (mm)	3.3 ± 0.4	3.3 ± 0.5	0.930
**Superior mesenteric arteriosclerosis**	16 (72.7%)	13 (50.0%)	0.143
**Inferior mesenteric arteriosclerosis**	16 (72.7%)	11 (42.3%)	**0.044**
**Mesenteric venous thrombosis**	1 (4.5%)	0 (0%)	0.458

CT, Computed Tomography; PI, pneumatosis intestinalis. The bold values indicating P value < 0.05.

### Endoscopic features and pathologic findings

**Figure 2:**
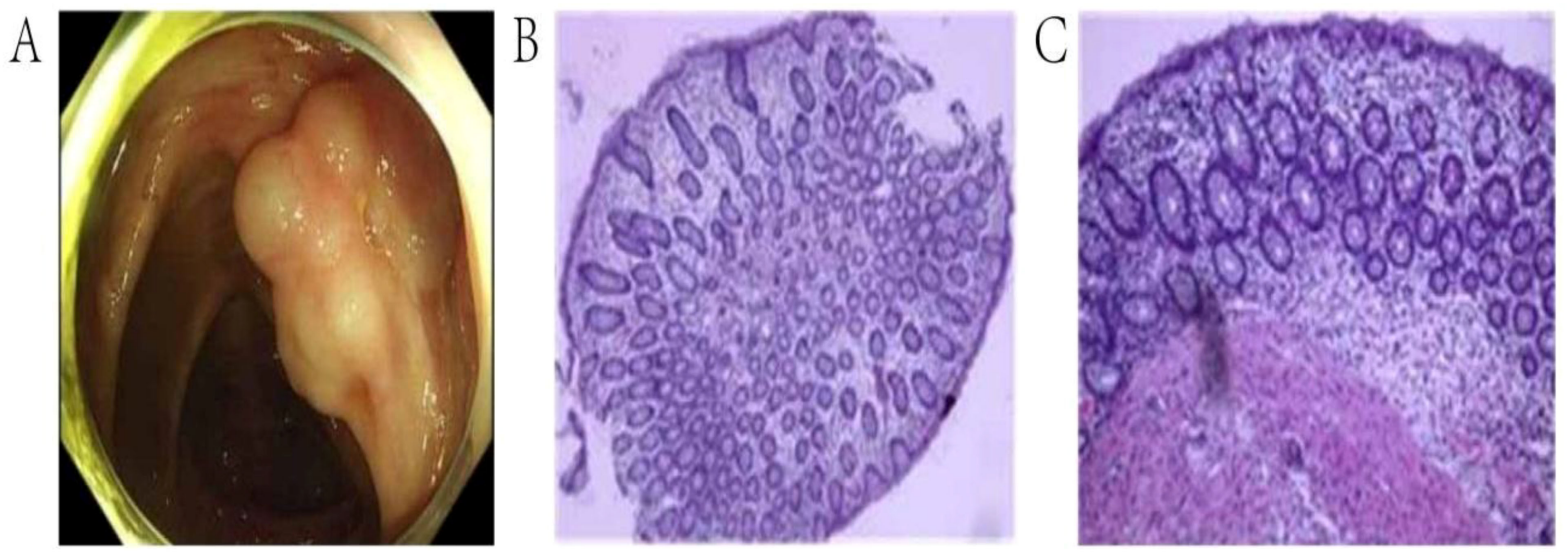
Endoscopic and pathological manifestations of pneumatosis intestinalis. **(A)**. Colonoscopy showed irregular submucosal cystic lesions in the ascending colon. **(B, C)**. The tissue biopsy of the lesion site revealed submucosal cystic structure.

Colonoscopy demonstrated diffuse submucosal cystic lesions in PI patients (**Figure 2A**). Infiltration of immune cells such as plasma cell and neutrophils and granulomatous inflammation can be seen in biopsy of the lesion site (**Figures 2B, C**).

### Treatments and clinical outcomes

For PI, conservative treatments such as anti-inflammatory, fasting water and food, gastrointestinal decompression, nutritional support, regulating intestinal microbiota, and acid suppression were adopted. Seven patients with intestinal ischemia performed surgical treatment. There was no significant difference in the treatment plan selection, hospitalization time, and clinical outcomes of patients taking or not taking acarbose (**Table 4**).

**Table 4:** Comparison of treatment and outcome of patients with pneumatosis intestinalis between the acarbose taken and untaken group.

Parameters	Acarbose taken (n=22)	Acarbose untaken (n=26)	P value
**Treatment**			0.687
Conservative therapy	18 (81.8%)	23 (88.5%)	
Surgery	4 (18.2%)	3 (11.5%)	
**Length of hospital stay** (days)	12 ± 7	11 ± 6	0.591
**Outcome**			0.483
Death	0 (0%)	2 (7.7%)	
Recovery	22 (100%)	24 (92.3%)	

## Discussion

In this study, we compared the clinical characteristics of patients who received acarbose with those who did not. Our findings indicate that PI induced by αGI predominantly occurs in older diabetic patients with underlying cardiovascular disease. PI patients who took αGI exhibited a higher likelihood of developing inferior mesenteric arteriosclerosis compared to those who did not utilize αGI.

Alpha-glucosidase is an enzyme that plays a crucial role in digestion by breakdown complex carbohydrates, such as starch and sucrose, into simple sugars (18). By inhibiting alpha glucosidase, the absorption rate of carbohydrates in the digestive tract can be reduced, slowing down the release of glucose into the bloodstream and thus reducing the sharp rise in postprandial blood glucose levels (19, 20). This mechanism is particularly beneficial for individuals with type 2 diabetes, as it aids in glycemic control without causing significant insulin release (21). Overall, αGI contribute to better overall blood glucose management in diabetic patients.

Acarbose is the main oral medication that clearly inhibits alpha glycosides at the brush border of the small intestine (22). It may be considered for use earlier in the elderly and/or when contraindications to metformin are present (23). The most important limiting factor for the dosage of acarbose is various common gastrointestinal side effects (23–25). In addition, its use is more common in Asian countries, where such drugs exhibit higher efficacy, possibly due to the carbohydrate rich eastern diet (26).

The pathogenesis of PI remains unclear. However, six pathophysiologic mechanisms have been proposed including inflammation, physical damage of intestinal mucosa, nutritional imbalance and dysbacteriosis, gastrointestinal dysmotility, and immune dysfunction (1, 27). The acarbose induced PI is caused by abdominal distention and increased flatus with the increased levels of intestinal gas (28). Because acarbose has the potential to inhibit alpha amylase, the unhydrolyzed starch that reaches the colon is easily fermented (2, 29). This mechanism is attributed to the fermentation of gut bacteria, which form metabolic products such as hydrogen, methane, and carbon dioxide from undigested carbohydrates (30). Some patients with diabetes will choose maltose instead of sucrose in order to make their blood sugar response less obvious, but maltitol and other indigestible sugar substitutes are easy to be metabolized into carbon dioxide and hydrogen through intestinal bacteria fermentation, resulting in PI (31).

PI can be found incidentally in asymptomatic patients, while some cases presented as abdominal pain, diarrhea, abdominal distention, constipation, bloody stool, flatus, loss of appetite, weight loss, and even life-threatening illnesses including bowel necrosis and perforation (32). Our study found that PI patients who took acarbose had a higher incidence of vomiting symptom, possibly due to increased intestinal gas levels caused by using alpha glycosidase inhibitors. Other gastrointestinal symptoms were common symptoms of PI.

The occurrence of PI could be in any age group and anywhere within the gastrointestinal tract from the esophagus to the rectum. It was previously reported that mainly involve the terminal ileum, but Morris later reported that PI localized to large intestine in 46% of the cases, the small intestine in 27% of the cases, the stomach in 5% of the cases and the large combined with small intestine in 7% of the cases (33). PI of the patients included in our study can occur in different parts of the digestive tract, such as ascending colon, transverse colon, descending colon, sigmoid colon, jejunum, ileum, duodenum, small intestine, esophagus, stomach wall, etc., and the imaging manifestations of the digestive tract of most patients are multiple PI. PI can occur in the wall, serosa, and mucosa of the intestinal wall, and in most patients, PI manifests as invading various levels of the intestinal wall.

In addition, our study also found that higher incidence of arteriosclerosis in the inferior mesentery in older patients with cardiovascular disease who take acarbose. This result can be interpreted as that patients taking acarbose are older and that diabetes itself is related to atherosclerosis. We should pay more attention to whether PI will occur after taking αGI for older diabetes patients with cardiovascular disease, which has a potential guiding role for drug treatment of diabetes patients in the future.

Absolutely, improving from the source of drug manufacturing to reduce the occurrence of complications more effectively is the most important part. Natural products derived from plants are a valuable source of therapeutic agents with minimal toxicity and side effects (34). Flavonoids, important ingredients in human diet, are phenolic compounds widely distributed in the plant kingdom, which show promising anti diabetes activities, including inhibition of alpha amylase and alpha glucosidase (35). Extracts or isolated forms of bioactive compounds have become feasible options for controlling hyperglycemia without side effects, but they still need to be used in combination with acarbose (36).

It is important to note that when using αGI, concerns regarding the potential discontinuation of αGI due to side effects should not be a significant issue, as other pharmacological options are also available that can effectively manage diabetes. There are several types of medications available for diabetes treatment in addition to αGI, each targeting different mechanisms to help control blood sugar levels (37). Biguanides, such as metformin, are commonly prescribed to improve insulin sensitivity and reduce glucose production in the liver (38). Sulfonylureas, like glipizide, stimulate the pancreas to release more insulin (39). DPP-4 inhibitors, such as sitagliptin, enhance the body’s incretin hormones to increase insulin production and decrease glucagon secretion (40). SGLT2 inhibitors, including empagliflozin, work by preventing glucose reabsorption in the kidneys, promoting its excretion through urine (41). Lastly, insulin therapy remains essential for those with type 1 diabetes and some with type 2, providing a direct method to control blood glucose levels (42). Each class of medication has its unique benefits and potential side effects, allowing healthcare providers to tailor treatment based on individual needs.

Several limitations should be acknowledged in this study. Firstly, it was a single-center, retrospective analysis, which may introduce inherent biases. Secondly, the sample size is relatively small, a challenge that arises from the rarity of PI, making it difficult to obtain a larger cohort. Thirdly, our study did not investigate the relationship between certain characteristics of individuals in different regions, such as dietary habits, and the occurrence of PI, which are related to diabetes and PI. However, the subjects included in our study are mainly local patients with largely similar dietary habits, which minimizes their impact on the results. Nevertheless, this remains a valuable research direction that can support future multicenter and multifactor analyses.

## Conclusions

Increased awareness of the potential for PI in older diabetic patients with cardiovascular diseases following αGI therapy is essential, and timely intervention is crucial to prevent adverse outcomes. Our study provides valuable insights for the use of αGI in diabetes treatment and suggests the need for further research to explore long-term monitoring and management strategies for patients at risk.

## Data Availability

The raw data supporting the conclusions of this article will be made available by the authors, without undue reservation.
